# Antimicrobial effect of phytosphingosine in acrylic resin

**DOI:** 10.1590/0103-6440202305357

**Published:** 2023-10-27

**Authors:** Luiza Sanchez Pejon, Viviane de Cássia Oliveira, Ayodele Alves Amorim, Júlia Correa Raffaini, Carolina Noronha Ferraz de Arruda, Fernanda de Carvalho Panzeri Pires-de-Souza

**Affiliations:** 1 Department of Dental Materials and Prosthodontics Ribeirão Preto School of Dentistry - University of Sao Paulo. Av do Café s/n, 14040-904 Ribeirão Preto, SP, Brazil.; 2 Department of Prosthodontics of School of Dentistry - Rio de Janeiro State University. Boulevard 28 de Setembro, 157, 20551-030 Rio de Janeiro, RJ, Brazil.

**Keywords:** color stability, anti-adherence, cell viability, phytosphingosine, sodium percarbonate

## Abstract

This study evaluated color stability (CS), anti-adherence effect (AAE), and cell viability of microorganisms on acrylic resin (AR) surface, treated associated or not with sodium percarbonate (SP). AR specimens were prepared, and color analysis was performed before and after the treatments and the CS was calculated. For analysis of AAE, the samples were sterilized by radiation in a microwave oven. Then samples were randomly distributed: phosphate-buffered saline (PBS - control), 0.5% sodium hypochlorite (SH), phytosphingosine (PHS), and phytosphingosine + SP (PHS+Na_2_CO_3_). The specimens remained in contact with solutions for 30 minutes and were later contaminated by *Candida albicans*. Aliquots were seeded in Petri dishes with Sabouraud Dextrose agar and incubated at 37°C for 24 hours. After the incubation, the number of colonies was counted. The cell viability of adhered microorganisms on the AR was evaluated and 20 fields were observed under an epifluorescence microscope, and the percentage of adhered viable cells was calculated. Data were compared (One-way ANOVA, Tukey, p<.05). As for CS, PHS+ Na_2_CO_3_ (0.4±0.1) resulted in less change than PBS (0.9±0.2), similar to the other groups (SH [1.0±0.3)]; PHS [0.9±0.2)]). There was no difference for all tested solutions regarding the ability to avoid microorganism adherence (p>0.05), but PHS (11.2±4.1) resulted in a smaller area of adhered viable cells, statistically different from SH (18.2±7.6) and PBS (26.4±10.8). It was concluded that PHS resulted in lower adhered viable cells and when associated with Na_2_CO_3_, also shows a lower effect on the CS of AR.

## Resumo

Este estudo avaliou estabilidade de cor (EC), efeito antiaderente (EAA) e viabilidade celular de microrganismos em superfície de resina acrílica (RA), tratada com solução de fitoesfingosina, associada ou não ao percarbonato de sódio (PS). Espécimes RA foram preparados e análise de cor foi realizada antes e após os tratamentos e EC foi calculada. Para análise de EAA, as amostras foram esterilizadas por radiação em forno de micro-ondas. Então foram distribuídas aleatoriamente: solução salina tamponada com fosfato (PBS - controle), hipoclorito de sódio 0,5% (SH), fitoesfingosina (PHS) e fitoesfingosina + SP (PHS+Na_2_CO_3_). Os espécimes permaneceram em contato com as soluções por 30 minutos e posteriormente foram contaminados por *Candida albicans*. Alíquotas foram semeadas em placas de Petri com ágar Sabouraud Dextrose e incubadas a 37°C por 24 horas. Após a incubação, o número de colônias foi contado. A viabilidade celular dos microorganismos aderidos na RA foi avaliada e 20 campos foram observados em microscópio de epifluorescência, e a porcentagem de células viáveis aderidas foi calculada. Os dados foram comparados (One-way ANOVA, Tukey, p<0,05). Quanto a EC, o PHS+ Na_2_CO_3_ [0,4 (0,1)] resultou em menor alteração que o PBS [0,9 (0,2)], semelhante aos demais grupos (SH [1,0 (0,3)]; PHS [0,9 (0,2)]). Não houve diferença para todas as soluções testadas quanto à capacidade de evitar a aderência de microorganismos (p>0,05), mas o PHS [11,2 (4,1)] resultou em uma área menor de células viáveis aderidas, estatisticamente diferente do SH [18,2 (7,6)] e PBS [26,4 (10,8)]. Concluiu-se que o PHS resultou em menor número de células viáveis aderidas e, quando associado ao Na_2_CO_3_, também apresenta menor efeito sobre o EC da RA.

## Introduction

To prevent and reduce the risks of local and systemic infections that compromise the health of denture wearers the correct hygiene and maintenance of these appliances have been increasingly recognized due to their importance in maintaining the intra- and extra-oral health of patients ^1^. Among local infections, prosthetic stomatitis is one of the most commonly present diseases and corresponds to an inflammation that affects the oral cavity with mucosal lesions, usually associated with the presence of *Candida albicans*, and can be potentiated in the presence of *Streptococcus mutans* ^2^. The presence of pores on acrylic resin, the material used for the denture base, represents one factor that facilitates microbial colonization and consequently the development of these diseases ^3^.

The most used methods for denture cleansers are the mechanical and chemical methods, and the association between them has been the most indicated ^4^. The mechanical method corresponds to brushing, preferably with a toothbrush and a specific toothpaste for dentures, with low abrasiveness ^4^. Chemical methods, on the other hand, correspond to the use of auxiliary solutions for disinfection, such as sodium hypochlorite, peracetic acid, and alkaline peroxide. They are usually used by immersing them for a short period (3-20 minutes) or overnight (8 hours) ^5^.

The use of sodium hypochlorite is common to patients and considered the gold standard among hygiene solutions; however, this solution must be used in low concentrations and for short immersion periods in order not to cause damage to the acrylic resin properties, such as color change and decreased flexural strength ^6^. Another solution highly recommended and used, is the use of effervescent tablets for cleaning dentures, such as sodium perborate and sodium percarbonate for short and long immersions ^7^ *.* Such compounds have shown good results when used according to the manufacturer's indication, showing a powerful hygiene action, since, when in contact with water, it releases oxygen and, thus, eliminates the anaerobic microorganisms from the oral cavity. Its efficiency turns even better if associated with the mechanical method, capable of removing microorganisms adhered to the denture base ^8^ *.*

The dentistry field has been interested to find a component that can reduce microbial adhesion on substrates and does not cause damage to the properties of acrylic resin, such as color changes. Phytosphingosine (PHS), is an agent present in the human body and acts in the defense of the organism. In addition, it is a lipid that has been used in several commercial situations, including cosmetics. A recent study has shown that PHS presents properties of anti-adherence of microorganisms on hydroxyapatite disks, avoiding the formation of biofilm, by creating a film pellicle that prevents the adhesion of these structures ^9^ *.*

According to Cukkemane *et al* ^9^, the positive portion of the phytosphingosine molecule is bound to the hydroxyapatite, and the negative portion remains available and performs an anti-adherent and antimicrobial function ^9^.

Another study demonstrated its bactericidal effect, preventing the proliferation of bacteria such as *S. mutans*, one of those responsible for stabilizing the biofilm formed by *Candida* spp., microorganisms related to prosthetic stomatitis ^2^ *.* In addition, PHS was also able to prevent *C. albicans* adhesion and salivary biofilm formation ^10^ *.* PHS solution also promoted a lower level of staining when subjected to cigarette smoke and resulted in an increase in the microhardness of the dental enamel ^11^.

There are no reports in the literature on the use of PHS as a chemical method of preventing bacterial colonization on denture bases. However, its properties are promising against microorganisms related to local infections in denture wearers, such as denture stomatitis ^1^. In addition, because it is a solution, there is the possibility that it can be used not only as therapy but also as a vehicle for other drugs, in this case, sodium percarbonate, to release oxygen and decrease the colonies of microorganisms.

Considering the protective properties of phytosphingosine and the chemical/mechanical action of sodium percarbonate, this study aimed to evaluate these solutions, associated or not, regarding the color stability, microbial anti-adherence effect, and the presence of viable cells on acrylic resin. For color stability, the null hypothesis was that there would be no difference in the color change of acrylic resin regardless of the hygiene solution tested. Regarding the anti-adherence effect, the null hypothesis was that PHS, associated or not with sodium percarbonate, would show the same anti-adherence ability on the surface of the material.

## Materials and methods

### Sample preparation

A pilot study estimated a priori sample size of n=6 (power=80%; α=5%). Acrylic resin samples (Classico, Artigos Odontologicos Ltda., Campo Limpo Paulista, Sao Paulo, Brazil) were obtained by the lost wax process. The wax was heated and filled into an aluminum metal matrix (15 x 3 mm), the excess was removed and then, samples were embeded (MAC - Artigos Odontologicos e Protese Ltda., Sao Paulo, Brazil) with type IV plaster (Gesso-Rio, Orlando Antonio Bussioli-ME, Rio Claro, Sao Paulo, Brazil).

The molds were cleaned by applying boiling water and the acrylic resin was handled according to the manufacturer's instructions, until establishing a final pressure of 1200 Kgf (Protecni-Protecni equip. med., Araraquara, Sao Paulo, Brazil), and polymerized by immersion in water (73ºC) for 90 minutes and boil for 30 minutes (Termocycler T100, Ribeirao Preto, Sao Paulo, Brazil).

The acrylic resin excess was eliminated and both flat faces of each sample were polished in the horizontal lathe spindle (DP9; Copenhagen, Denmark) with 220/400/600/1200-grit silicon carbide paper (Norton Saint-Gobain do Brazil Prod.Ind., Guarulhos, Sao Paulo, Brazil). Samples were accepted and included in the study with a maximum of 0.30 µm of surface roughness ^12^.

A total of 58 samples were obtained (n=6), 34 to evaluate the solutions’ anti-adherence effect and cell viability for tested solutions and microorganisms controls, and another 24 were used for the analysis of the color stability.

### Color Stability

Color stability was determined by a colorimeter (Color Guide 45/0, BYK Gardner GmbH, Geretsried, Germany), initial (baseline) and final (after treatments) color readings were performed against a white background (White Standard Sphere for 45°, 0° Reflectance, and Color Gardner Laboratory, Germany) and inside a standardized lightbox (Gester International, Fujian, China) with a neutral-gray (Munsell N-7) background and a standard illuminant D65, which simulates the light spectrum of the day.

The black-white (L*), green-red (a*), and blue-yellow (b*) color dimension values were measured by the CIELab color system on each specimen and the data were considered as initial color measurements (Baseline).

After the initial readings, the samples were randomly distributed: PBS: Phosphate Saline Buffer (control); SH: 0.5% Sodium Hypochlorite (Inject Center, Ribeirao Preto, Sao Paulo, Brazil); PHS: phytosphingosine solution (5mg/mL); PHS+Na_2_CO_3_: Sodium Percarbonate dissolved in PHS. Each sample was subjected to the treatments 2x a day, for 30 minutes immersion in cleanser solution, for 7 days, to simulate 14 days of use. Between applications of each protocol, the samples were rinsed with running water and stored in artificial saliva until the next cycle.

After the treatments, the final measurements were performed. The color change (ΔE_00_) was calculated using the formula ^13^: ΔE_00_ = (ΔL/K_L_ . S_L_) + (ΔC/K_C_ . S_C_)^2^ + (ΔH/K_H_ . S_H_)^2^ + RT. (ΔC/K_C_ . S_C_) X (ΔH/K_H_ . S_H_) ^0.5^, where ΔL’, ΔC’, and ΔH’ are the differences in lightness, chroma, and hue, respectively, between two measures and RT (rotation function) is a function that accounts for the interaction between chroma and hue differences in the blue region. S_L_, S_C_, and S_H_ are the weighting functions for the lightness, chroma, and hue components, respectively; and K_L_, K_C,_ and K_H_, are the parametric factors according to different viewing parameters that were set to 1 ^14^.

The data were then quantified according to the National Bureau of Standards units (NBS) ^15^ using the following formula: NBS units = ΔE × 0.92. The data were then classified according to the following: 1) trace: 0.0-0.5; 2) slight: 0.5-1.5; 3) noticeable: 1.5-3.0; 4) considerable: 3.0-6.0; 5) very: 6.0-12.0; 6) excessive: >12.0.

### Protective ability on microbial adhesion

The ability of solutions to protect the microorganism’s adherence was evaluated in duplicate, in two independent time intervals (n=3). After roughness standardization, sterilized by microwave irradiation [127V, 800W, 2,450 MHz (Perfect; Panasonic, Kadoma, Japan), at 650 W for 6 min ^16^ ^)^ were aseptically distributed into four groups according to the hygiene protocol used.

According to Bikker *et al*. ^10^, the minimum inhibitory concentration (MIC) of phytosphingosine in salivary inoculum is 37.5 µg/mL. Thus, in the present study, the PHS solution was used at a final concentration of 100 µg/mL, i.e., about 2.7 times the MIC, and was prepared in ethanol at a concentration of 5 mg/mL and diluted in 20 mM Tris supplemented with 0.1% Tween 20 (pH 6.8), to keep the PHS in solution ^9^ ^,^ ^10^ ^,^ ^16^ ^,^ ^17^.

PHS+Na_2_CO_3_ was formulated from the dissolution of 68.635% in weight ^(^ ^18^, by adding 2.68g of sodium percarbonate to (300 mL) of phytosphingosine, following manufacturers of Effervescent Tablets instructions (Corega Tabs; GlaxoSmithKline, Middlesex, United Kingdom).

Each sample was transferred to a polyethylene tube containing the hygiene solutions (5mL) and was taken to the incubator with 75rpm agitation at 37ºC, where they remained for 30 minutes. After this period, the samples were transferred to Petri dishes, where 3 sequential rinses were performed to remove residues from the hygiene solutions. They were then contaminated and seeded with *C. albicans*, with Sabouraud Dextrose Broth and Agar respectively (HiMedia Laboratories Pvt. Ltda., Mumbai, Maharashtra, India).

*C. albicans* (ATCC 10231) was plated in Sabouraud dextrose broth (SD) for 18 to 24h at 37ºC. Then, the samples were centrifuged at 4200g for 5 minutes in a 5430R Centrifuge (Eppendorf AG, Hamburg, Germany). The supernatant was discarded, and the volume was made up to 10mL with PBS. The cells were homogenized and centrifuged again. The cell pellet was washed twice.

To standardize the *C. albicans* inoculum, the microorganisms were added to the PBS solution and the Neubauer chamber was used, due to the variable morphology of the genus. Then, inoculation of the culture medium was performed at a concentration of 1×10^6^ CFU/mL (initial cell concentration for biofilm formation and adhesion assay).

Samples were aseptically distributed into 12-well tissue culture plates (TPP Techno Plastic Products, Trasadingen, Schaffhausen, Switzerland). Each well received 2 mL of medium broth containing standardized cell suspension (10^6^ CFU/mL) of *C. albicans*, except for the negative control group, which received 2 mL of sterile culture medium and was not contaminated.

The plates were incubated at 37°C, at 75 rpm for 1 h and 30 minutes (adhesion phase) and then rinsed twice with PBS. Then, 2 mL of sterile culture medium was added to each well, and the plates were incubated again for 48h to promote biofilm maturation ^19^. After 48 h of incubation, the samples were rinsed to remove plankton cells and were transferred to test tubes containing 10 mL of PBS, and sonicated (200W, 40KHz) (Altsonic Clean; Alt equipamentos, Ribeirao Preto, Sao Paulo, Brazil) for 20 min. Serial dilutions aliquots (10^1^ - 10^3^) of the resulting suspension were seeded, the number of colonies was registered, and the CFU/ml value was calculated by the formula: CFU/mL = number of colonies x 10 ^n^/q, where n: absolute value of the dilution (0, 1, 2, or 3) and q: volume, in mL, of the colony pipetted to each dilution at seeding (0.05 mL).

### Cell viability

The presence of viable cells was determined using the LIVE / DEAD^TM^ Biofilm Viability Kit (Molecular Probes, Inc., Eugene, Oregon, United States of America). Two samples were distributed into 12-well tissue culture plates with 2 mL of inoculated culture medium at a concentration of 1×10^6^ CFU/mL. The samples were rinsed in PBS and stained for 15 minutes, LIVE/DEAD Kit was applied and evaluated after 2 h of incubation ^9^ by an epifluorescence microscope (EM) (Carl Zeiss, Axio Observer A1, Jena, Thuringia, Germany) inverted with appropriate filters and a magnification of 400×.

Twenty images were obtained at random locations for each sample. All images were analyzed by ZEN 2.3 lite software (Carl Zeiss) to measure the area of the image covered by cells. The results were presented in (%) according to the total image area ^20^.

### Data analysis

The values obtained from the analyses were submitted to appropriate statistical tests with the help of a statistical program (GraphPad Prism 8.0.1). The data were evaluated for normality by the Shapiro-Wilk test (α=0.05), which resulted in normal distribution for all variables. Thus, statistical analyses were performed using One-way ANOVA, followed by Tukey's test, with a power of 80% and a significance level of 95%.

## Results

### Color Stability

**Figure 1:**
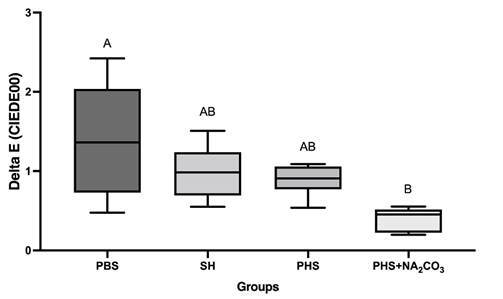
Comparison of the color stability

Figure 1 shows the ∆E_00_ comparison of means (One-way ANOVA, Tukey, p<0.05). Regarding color stability, it was possible to verify that the smallest change occurred after treatment with PHS+NA_2_CO_3_, different statistically from PBS, which showed the greatest color change and resulted in no difference from the other groups. PHS+ Na_2_CO_3_ (0.37) resulted in the smallest NBS values, classified as trace, while the other solutions were classified as slight (PBS: 1.29, SH: 0.92, PHS: 0.83), which, according to the NBS classification, is not clinically significant.

## Protective ability on microbial adhesion

The values of *C. albicans* CFU/mL count, after the use of the hygiene solutions, were transformed to log10 ^(UFC+1),^ and the means were statistically analyzed using the One-way ANOVA test, Tukey, p<0.05. The comparison of means can be seen in Table 1. The statistical analysis showed that there was no statistically significant difference between all groups (p>0.05).

**Table 1:** Comparison of means (standard deviation) of CFU/Ml and biofilm area (%).

	PBS	SH	PHS	PHS+NA_2_CO_3_
CFU/mL	3.0 (0.4)	3.5 (0.4)	3.3 (0.1)	3.1 (0.7)
Biofilm area (%)	26.4 (10.8) A	18.2 (7.6) B	11.2 (4.1) C	13.7 (6.6) BC

For CFU/mL comparisons p>0.05. For the biofilm area, different letters indicate a statistically significant difference (p<0.05). PBS: Phosphate Saline Buffer; SH: 0.5% Sodium Hypochlorite; PHS: phytosphingosine solution (5mg/mL); PHS+Na_2_CO_3_: Sodium Percarbonate dissolved in PHS.

## Cell viability

**Figure 2:**
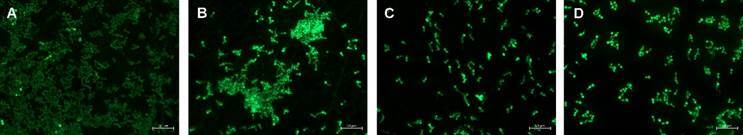
Viable cells after treatments with the hygiene solutions; a) PBS; b) SH; c) PHS; D) PHS+Na_2_CO_3_. The higher presence of viable cells for PBS and the lowest for PHS statistically different (p<0.05) from the SH group, PHS+ Na_2_CO_3_ showed intermediate results.

Regarding cell viability, the mean of viable cell covered area (%) was measured and is shown in (%), which was analyzed according to One-way ANOVA, Tukey, p<0.05 (table 1). Figure 2 shows the epifluorescence microscope images with the analysis of cell viability.

After treatment with PBS, there was a higher presence of viable cells, statistically different from all other groups treated with hygiene solutions. The lowest cell viability occurred after PHS treatment, statistically different (p<0.05) from the SH group. The group treated with PHS+ Na_2_CO_3_ showed intermediate results.

## Discussion

The study objective was to evaluate the color stability, the solutions’ ability to protect the acrylic resin surface from microbial adhesion, and the cell viability after using solutions of phytosphingosine, associated or not with sodium percarbonate.

Regarding color stability, the null hypothesis was rejected, since PHS+Na_2_CO_3_ resulted in less color change than PBS (control solution). The color changes can be explained by the plasticization of the polymer chains ^(^ ^21^, that is, the relaxation of the polymeric chains and their distancing can cause changes in the reflection index of the resin, resulting in a change in the final color.

Literature shows that sodium hypochlorite solutions and sodium perborate-based solutions produce color changes in acrylic resin, in a proportional way to the contact time of these solutions with the material ^6^ ^,^ ^7^. Thus, hygiene solutions that are antimicrobial effective in short immersion times are currently being sought, to avoid alterations in the properties of the resin over time ^5^ ^,^ ^7^.

In the present study, the resulting color changes were considered clinically accepted ^(^ ^22^, following NBS classification, for all solutions, and PHS+Na_2_CO_3_ showed the smallest color alteration. These small changes may have resulted from the treatment time applied and the period evaluated, data corroborated by Haghi *et al* ^23^. Other studies of short immersions, even evaluating prolonged periods such as 5 years, did not show significant color change after the use of denture cleansers ^7^ ^,^ ^8^ ^,^ ^24^.

Regarding the PHS solution, data showing its effects on color change is still scarce. Amorim *et al*. ^11^ evaluated the protective effect of phytosphingosine (PHS) against staining on dental enamel and found that it did not protect the staining of the enamel by coffee and tea but was also within the acceptable limit.

Regarding the minor color changes from PHS+Na_2_CO_3_, sodium percarbonate, when dissolved in water, generates hydrogen peroxide, which is a bleaching agent, and also releases oxygen gas, which mechanically removes the biofilm ^8^. However, in this study sodium percarbonate was dissolved in PHS solution in ethanol with a concentration of 5mg/mL. Percarbonate is also soluble in ethanol ^25^, which has greater cleaning power ^26^. When percarbonate dissolves in water, it forms an alkaline solution (34). According Amorim *et al*. ^11^, acidic solutions produce a greater color change. Thus, the percarbonate can cause less color change by forming an alkaline solution. Allied with this, the PHS involved in the solution has the action of forming a sphingolipid layer on the surface of the material, decreasing the bleaching action of the percarbonate ^9^.

Regarding the solutions’ ability in protecting microorganism’s adherence to the acrylic resin surface, the null hypothesis was accepted, since there was no statistically significant difference between the groups. However, when cell viability was evaluated, the null hypothesis was rejected, as the percentage of adhered viable cells was lower when PHS solutions were used. Previous studies reported PHS effect against Gram-positive and negative microorganisms on other surfaces ^28^.

Although bactericidal effect against Gram-positive and negative microorganisms has been reported in other types of surfaces ^28^, which were not performed yet on acrylic resin. This material has inherent porosity, which contributes to the dissemination of microorganisms on its surface and subsurface, working as a reservoir of microorganisms ^29^. In addition, PHS showed antibiofilm activity by forming a protective film in hydroxyapatite surfaces ^9^. However, this study showed that all tested solutions were not able to protect the acrylic resin surface from the adherence of microorganisms.

However, the culture medium used for contamination of the specimens (Sabouraud dextrose) is diluted in PBS and the contamination method provides for constant agitation of 75 rpm to ensure adhesion of the microorganisms on the samples. Thus, the absence of antimicrobial activity of the solutions may have resulted from the leaching of the treatment solution into the aqueous environment of the culture medium, enhanced by stirring the solution for 48h. These results are corroborated by other authors ^9^ ^,^ ^10^, which found the antibiofilm activity of PHS in static situations. Over prolonged periods of contamination, the effect of PHS appears to diminish due to the growth of primary and secondary colonies of microorganisms. It is important to highlight that as the solution was tested by its effects on the prevention of biofilm adhesion, this also occurred after treatment with sodium hypochlorite, considered the gold standard solution among denture cleansers, as the results obtained were not related to the antimicrobial action of the treatments ^3^.

In addition, biofilm control should not be limited to the evaluation of the solutions’ anti-adherence ability, but other evaluations such as antimicrobial action and cell viability should also be taken into consideration to consider the real action of the treatments performed.

According to previous research ^9^ ^,^ ^10^, PHS makes the surface where it is applied more fluid and dynamic. PHS molecules, amphiphilic with a hydrophobic tail and a hydrophilic radical, make the binding of the microorganism inefficient due to the rotation and movement of its molecules ^30^ so that the positive portion adheres to the surface of the material, leaving the hydrophobic portion free, decreasing the bond with the microorganism ^9^. The association of percarbonate with PHS seems to be irrelevant for antimicrobial adherence since its effect was similar to PHS used alone, that is, the beneficial effect was a result of the action of PHS and not of percarbonate.

Previous studies have reported that sodium hypochlorite is an effective agent in reducing *C. albicans* adherence both in vitro and in vivo ^6^ ^,^ ^16^. Thus, by resulting in a biofilm area similar to hypochlorite, PHS associated with percarbonate demonstrates that it has similar efficiency to hypochlorite.

Although brushing is still an efficient method for removing biofilm, the use of auxiliary solutions for denture bases is still very useful considering the characteristics of this material. However, they must act on microbial reduction without leading to adverse effects on the acrylic resin. In this context, after the analyses in this study, it is possible to verify that PHS, associated or not with percarbonate, was able to reduce the viable count of adhered microorganisms on the acrylic resin, without changing the color stability of the acrylic resin, making this new solution a viable product to be used as a protective treatment against biofilm adhesion. 

Regarding the clinical relevance, PHS, associated or not with Na_2_CO_3_, showed to be an interesting component to prevent biofilm adhesion. One limitation of the present research was the non-reproducibility of the oral environment which is mainly influenced by saliva. In addition, mixed microbial biofilms were not evaluated. Future studies should not only involve mixed and complex biofilm but also tests to evaluate the mechanism of action of the PHS solution regarding the acrylic resin.

Considering the findings of the study, it is possible to conclude that, in the proposed treatment, no solution was able to avoid microorganism adherence. Despite this, the PHS resulted in fewer viable cells adhering to the acrylic resin. When associated with sodium percarbonate, it presents similar efficiency to sodium hypochlorite without causing any immediate changes in the acrylic resin color stability.
